# Blood Culture Testing Outcomes among Non-Malarial Febrile Children at Antimicrobial Resistance Surveillance Sites in Uganda

**DOI:** 10.1017/ash.2025.310

**Published:** 2025-09-24

**Authors:** Rogers Kisame

## Abstract

**Background:** Globally, Antimicrobial Resistance is a growing threat to global health security and economic development. Due to multidrug resistance, bloodstream infections (BSI) are a growing public health concern and a common cause of morbidity and mortality, especially among non-malarial febrile children **Method:** This study assessed laboratory BC process outcomes among non-malarial febrile children below five years of age at five AMR surveillance sites in Uganda between 2017 and 2018. Secondary BC testing data was reviewed against established standards. **Result:** Overall, 959 BC specimens were processed. Of these, 91% were from female patients, neonates, infants, and young children (1-48 months). A total of 37 AMR priority pathogens were identified; Staphylococcus aureus was predominant (54%), followed by Escherichia coli (19%). The diagnostic yield was low (4.9%). Only 6.3% of isolates were identified. AST was performed on 70% (18/26) of identified AMR priority isolates, and only 40% of these tests adhered to recommended standards. **Conclusion:** Interventions are needed to improve laboratory BC practices for effective patient management through targeted antimicrobial therapy and AMR surveillance in Uganda. Further research on process documentation, diagnostic yield, and a review of patient outcomes for all hospitalized febrile patients is needed.

